# From Volvulus to Short Bowel Syndrome: The Impact of Extensive Gangrene

**DOI:** 10.7759/cureus.66817

**Published:** 2024-08-13

**Authors:** Rechal Jaiswal, Shlok A Jaiswal, Himanshu D Husukale, Himali Bendale, Sharvi R Chavan

**Affiliations:** 1 General Surgery, Jawaharlal Nehru Medical College, Datta Meghe Institute of Higher Education and Research, Wardha, IND; 2 General Surgery, Institute of Medical Sciences (IMS) and SUM Hospital, Bhubaneswar, IND; 3 Surgery, Jawaharlal Nehru Medical College, Datta Meghe Institute of higher Education and Research, Wardha, IND; 4 Surgery, Jawaharlal Nehru Medical College, Datta Meghe Institute of Higher Education and Research, Wardha, IND

**Keywords:** total parenteral nutrition, short bowel syndrome, intestinal obstruction, ileosigmoid knot, compound volvulus

## Abstract

Compound volvulus, also known as ileosigmoid knotting, is an unusual but dangerous surgical condition that causes intestinal obstruction. It is a rare condition when an area of the small intestines is twisted and interrupts the blood supply, a closed-loop obstruction that is not common for one of the causes of intestinal blockage. Still, it is essential to distinguish the difference between an ileosigmoid knot and a simple sigmoid volvulus from each other, which are managed differently. Unlike abdominal X-rays, which are often nothing but clear images, CT scans or MRIs in their place provide more precise diagnostic information to help this problem diagnosis be traced. The first step in treating the patient is to immediately do an emergency laparotomy if the case involves ileosigmoid knotting, and which specific surgical procedure to use-resectional or non-resectional-is determined by the surgeon during the operation, who is considering such scales as the extent of the affected vascular compromise, the presence of necrosis or perforation, and the overall viability of the involved intestinal loops to conduct the most sought-after therapy.

In this case, the 45-year-old male presented with insidious-onset abdominal pain, vomiting, and constipation, along with signs of dehydration and hypotension. Physical examination revealed a distended abdomen, absent bowel sounds, and elevated white blood count and lactate levels, with an erect abdominal X-ray showing a dilated bowel, suggesting acute bowel obstruction with possible ischemia. This clinical presentation is consistent with acute bowel obstruction, potentially due to ileosigmoid knotting, which requires urgent surgical intervention.

Short bowel syndrome is a malabsorptive disorder characterized by the presence of less than 200 cm of the small bowel, sometimes as a result of congenital or surgical causes. This is a real problem for an individual because he or she must be cautious and watch what they eat and how much they eat fortified foodstuffs, as the decreased absorptive capability of the small intestine can restrict the body's ability to take in and make use of necessary nutrients, fluids, and electrolytes.

## Introduction

Ileosigmoid knotting (ISK) is when the ileum, an important part of the small intestine, wraps around the base of the sigmoid colon, causing a blockage. This condition can result in a critical situation where the blood supply to the affected tissues is obstructed, causing ischemia and potential necrosis in the ileum, sigmoid colon, and occasionally the cecum and ascending colon [1,2]. ISK is more commonly reported in regions such as Africa, Asia, and the Middle East, where sigmoid volvulus is prevalent. Nonetheless, it remains less common in Western countries [3]. There are a few main factors that contribute to ISK, including having a long mesentery (which is the tissue that attaches the small intestine to the back of the abdominal wall), a small intestine that moves around freely, and a sigmoid colon that is elongated with a narrow base. Other possible causes may include a diet high in fiber when the small intestine is empty, being in the later stages of pregnancy, surgical adhesions, malrotation, internal hernias, and Meckel's diverticulum [4,5]. The clinical presentation of ISK includes symptoms such as rebound pain, nausea, vomiting, abdominal discomfort, distension, and the inability to pass feces or flatus. ISK can rapidly progress to hypovolemic and toxic shock in most patients due to fluid loss, absorption of toxins from intestinal obstruction, and bowel ischemia or gangrene [6]. These complications can lead to shock, peritonitis, and endotoxemia due to fluid loss, bacterial translocation, and toxin absorption. 

## Case presentation

**Figure 1:**
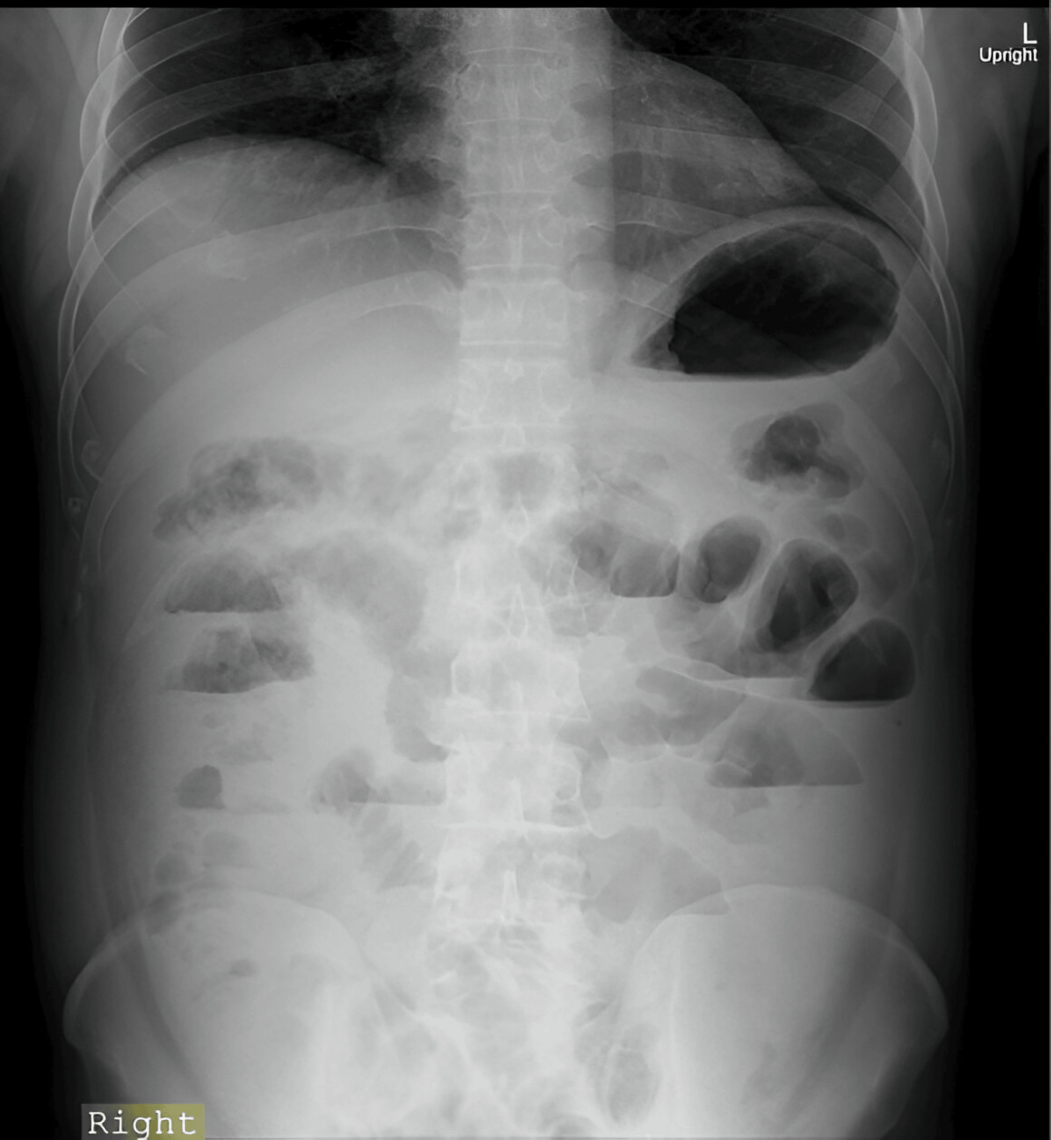
Abdominal X-ray showing multiple air fluid level

A 45-year-old male presented to the emergency department with complaints of abdominal pain, which was insidious in onset, gradually progressive, and colicky, along with a history of non-bilious, non-projectile vomiting and constipation for one day. On examination, he appeared distressed and dehydrated, with a blood pressure of 81/55 mmHg, a pulse rate of 124 beats per minute, a respiratory rate of 30 breaths per minute, and a temperature of 36.3°C. His abdomen was distended, showing widespread tenderness, guarding, and the absence of bowel sounds. A nasogastric tube was inserted for proximal decompression, and urethral catheterization was performed. A per-rectal examination showed a normal sphincter tone with fecal staining. Laboratory investigations revealed a white blood count of 20,400/cu mm (standard range: 4,000 to 11,000 cu/mm) and a lactate level of 36.1 mmol/L (standard range: -0.5 to 2.2 mmol/L). An erect abdominal X-ray revealed multiple air-fluid levels, as shown in Figure 1. 

**Figure 2:**
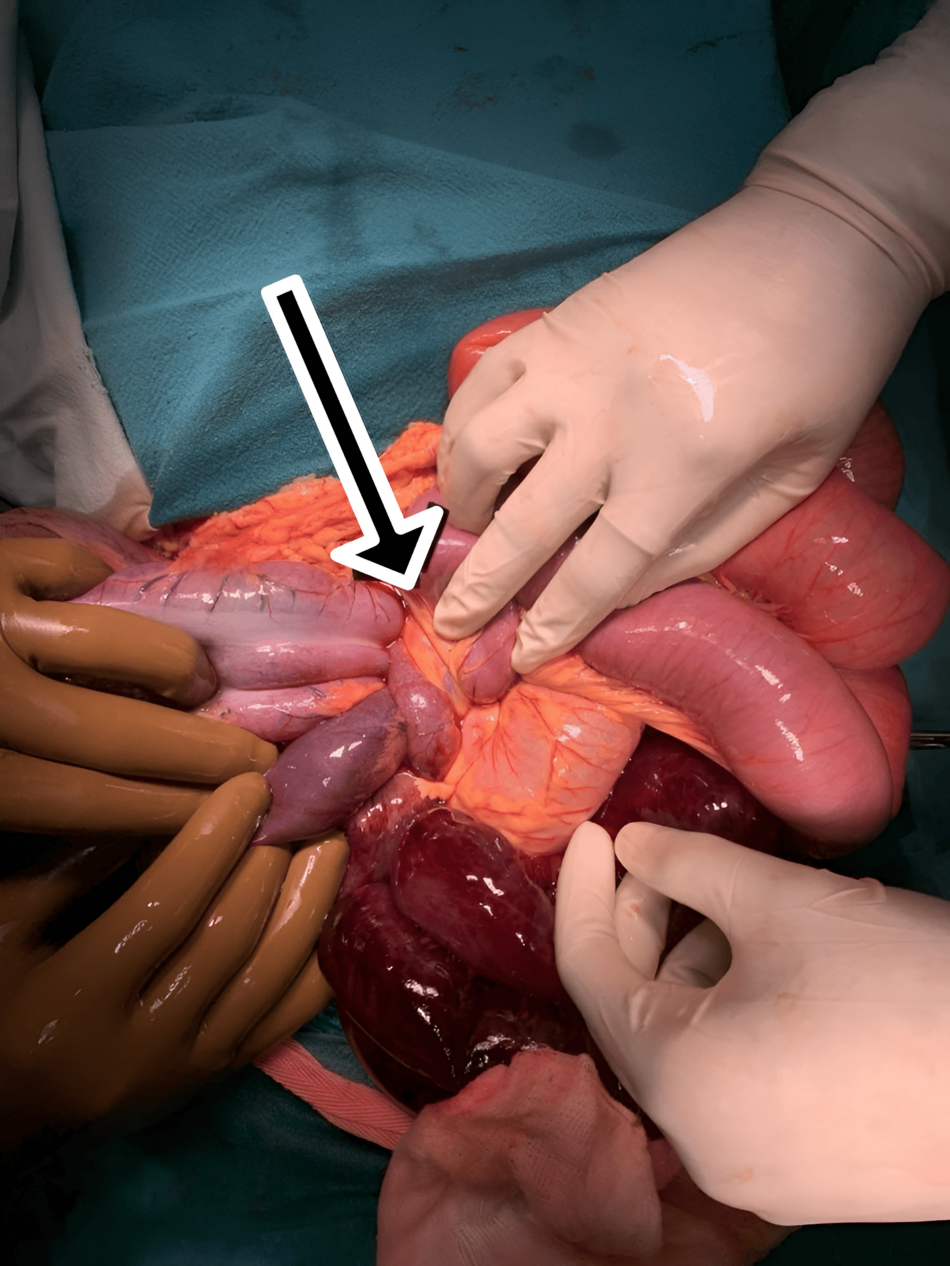
Intraoperative finding: distal ileum knotting around the base of the sigmoid volvulus loop

Contrast-enhanced computed tomography (CECT) taken at a local hospital (the picture was blurred) shows moderate ascites with closed loop obstruction that is suggestive of compound volvulus, distal ileum twisting (360) around the sigmoid colon, and its mesentery. The patient was given intravenous resuscitation fluids and a broad-spectrum antibiotic. Subsequently, he was rushed into the operating room for an emergency exploratory laparotomy, and a knotted distal ileum around the base of the sigmoid loop was revealed, as shown in Figure 2.

**Figure 3:**
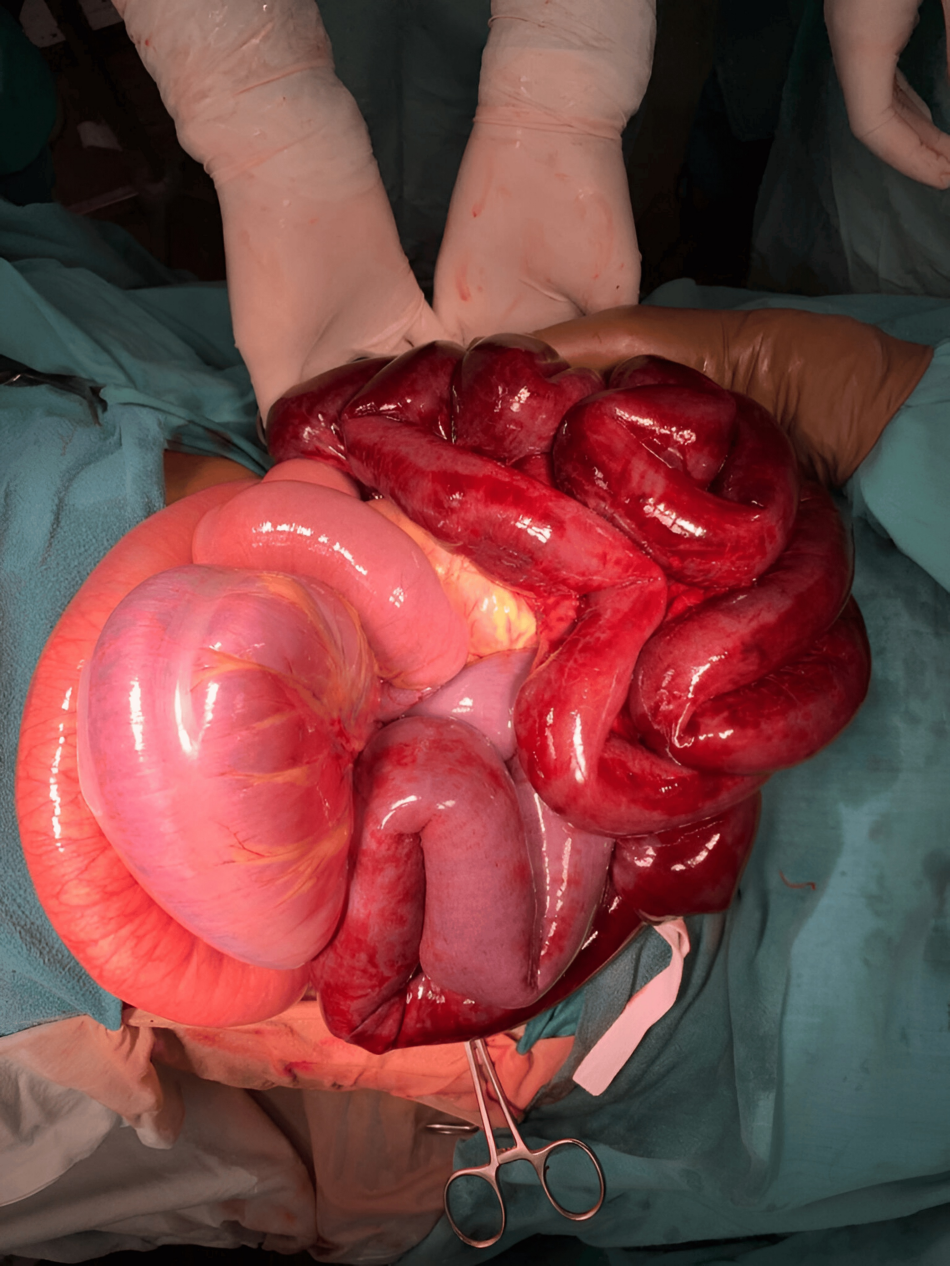
Intraoperative findings: extensive gangrene in the ileum, distal jejunum, and sigmoid colon

**Figure 4:**
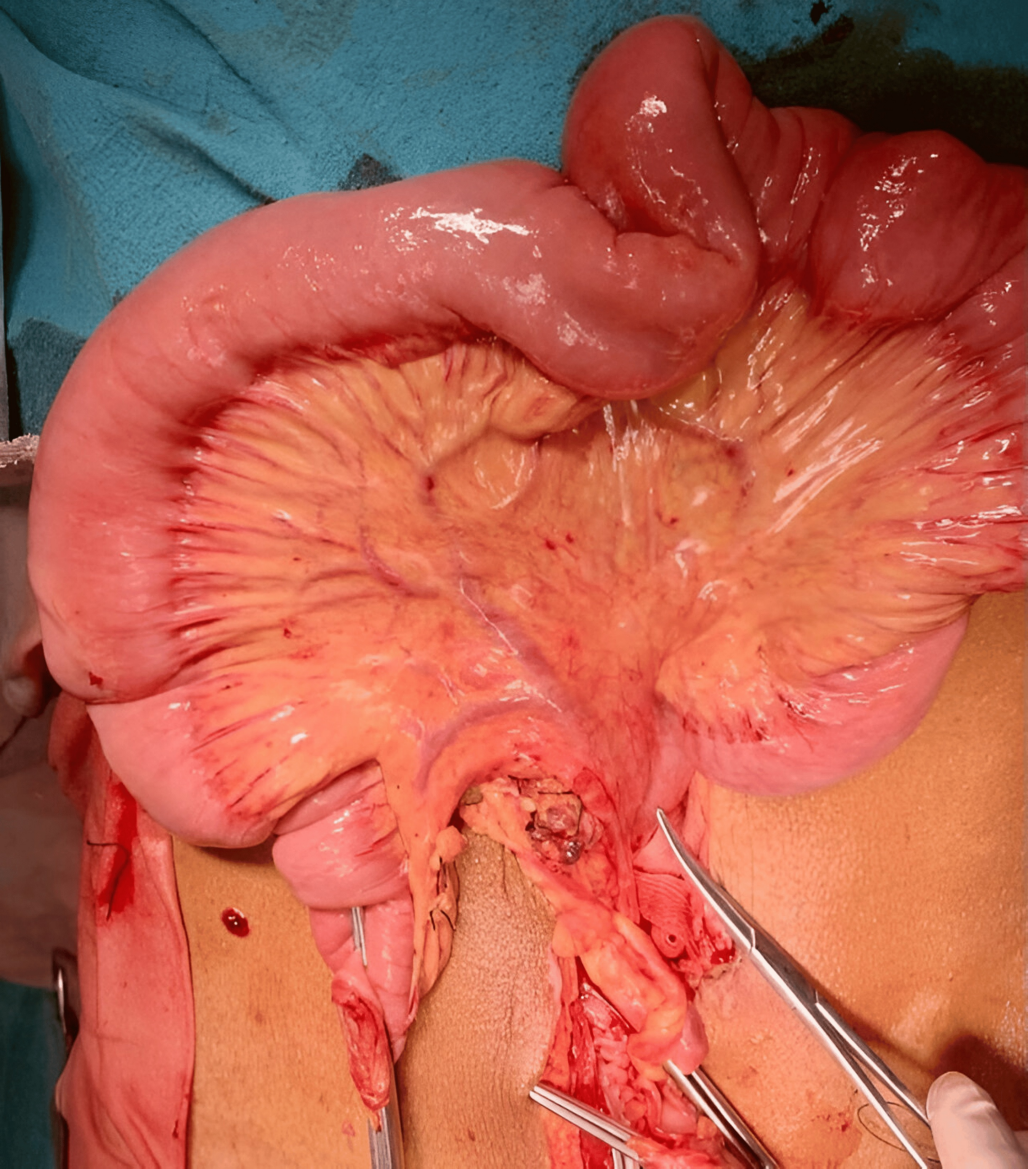
Remaining jejunum after resection of gangrenous distal jejunum, ileum to ileocecal junction, and sigmoid colon with appendicectomy and end jejunostomy

**Figure 5:**
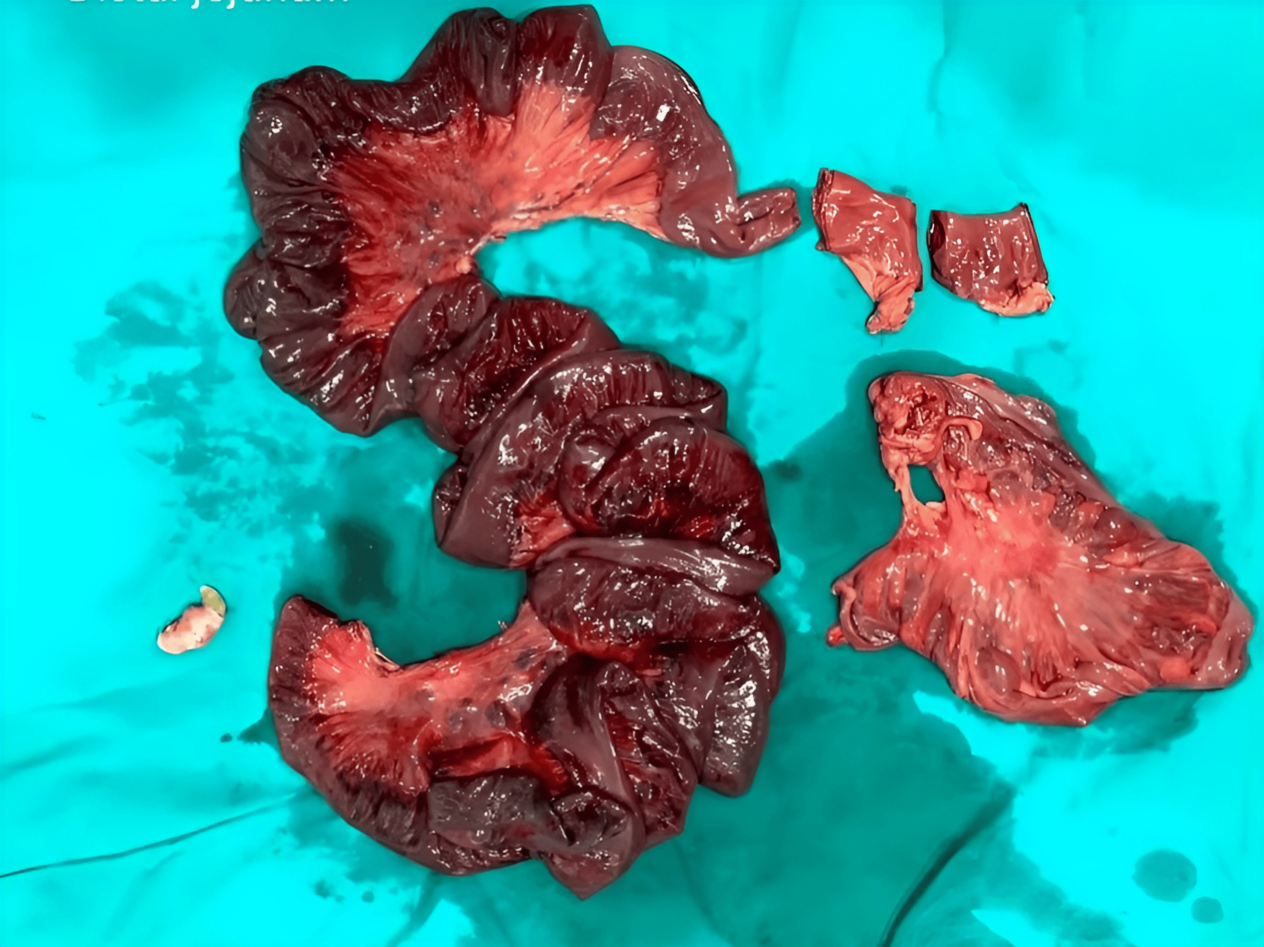
Resected specimen showing distal jejunum, entire ileum with mucocele, appendix, and sigmoid colon

During surgery, extensive gangrene involving the ileum, distal jejunum, and sigmoid colon was observed, as shown in Figure 3, along with the rotation of the small bowel around a fibrous band extending from the ileocecal junction to the sigmoid colon. Resection of gangrenous distal jejunum with whole ileum till ileocecal junction and sigmoid colon with appendicectomy with end jejunostomy was performed as shown in Figure 4. Following the resection of the sigmoid colon, the rectal stump was closed after ensuring there was no residual fecal matter or any material that could lead to a closed-loop obstruction. The cecal stump was also closed, and an end jejunostomy was created. The resected specimen of the distal jejunum, entire ileum with mucocele, appendix, and sigmoid colon is shown in Figure 5.

After surgery, the patient developed short bowel syndrome, which was managed with micronutrients and proper nutrition. Following an adequate gain in body weight, stoma reversal was performed with jejuno-colic anastomosis and colorectal anastomoses after three months. The patient was discharged after an eight-day hospital stay with no notable post-operative complications.

## Discussion

The development of the intestinal shaft volvulus has variegated causative factors, some of which include an elongated mesentery of a small bowel admitting a small bowel freely, a prolonged sigmoid colon with a narrow pedicle, and proper nutrition, like a high-fiber diet with an empty small bowel. Some tests make the suggestion that such a diet may cause the upper jejunum to be pushed faster and, thus, the small bowel to be more mobile so that it may enter or be found in the bottom compartment of the left lower abdomen [7]. The small bowel loop in this particular position is able to play a significant role in the rotation of the narrow sigmoid pedicle, and the peristalsis can, in turn, drive it to the extent of a closed loop of the intestine. Aside from these anatomical factors, the important links connecting some other factors, such as advanced pregnancy, trans-mesenteric herniation, Meckel's diverticulum volvulus, and ileocecal intussusception, also influence the pathogenesis. ISK primarily arises from specific groups of people in Africa, Asia, and the Middle East, and it's rather uncommon among whites [3]. Ileosigmoid knot (ISK) is hard to see during tests, as it is not something most people get to see very often. It can sometimes come off as merely being a simple sigmoid volvulus, like on X-rays, so a false diagnosis could be dangerous if one attempts an endoscopic decompression using a sigmoidoscope. There is a risk that a perforation or any injury might occur. The ISK fatality rate of 48% was the highest of them all [6].

Initial management of ISK involves ensuring first hemodynamic stability through aggressive intravenous fluid resuscitation and correction of acid-base imbalances. Surgical intervention is recommended once hemodynamic stability is achieved. Antibiotic therapy with cephalosporins and aminoglycosides is typically administered before and after surgery for 5-7 days, particularly metronidazole for anaerobic bacteria. Various surgical procedures are described in the literature and chosen based on intraoperative findings. The standard surgical approach for treating ileosigmoid knotting usually includes resecting the involved segments of the ileum and sigmoid colon with a primary enteroenteric anastomosis. However, if there is gangrene affecting both the ileum and sigmoid, more extensive procedures such as Hartmann's procedure or colostomy may be required for managing the descending colon. These procedures involve creating a stoma to redirect fecal flow and are important when bowel viability is compromised beyond the ileum and sigmoid. In a large single-center series, ISK had an incidence of 1.6 cases per year and 0.4 cases per 100,000 persons per year, with a mean age of 47.5 years and a predominance in males [4,5]. Common symptoms included abdominal tenderness upon palpation, abdominal distention, hypo/akinetic bowel sounds (reduced or absent bowel sounds), an empty rectum upon digital examination, guarding/rebound tenderness, hyperkinetic bowel sounds, and melanotic stool. Plain abdominal X-ray findings were evident in only a small percentage of patients, and the accurate preoperative diagnosis rate was low, often resulting in misdiagnoses such as non-specific intestinal obstruction or non-obstructive acute abdomen [8]. 

Our patient is male and of Asian origin, diagnosed with symptoms of peritonitis and described with abdominal pain. He initially received empirical antibiotics and intravenous fluids that helped decrease his symptoms. After hemodynamic stability was achieved in a patient, exploratory laparotomy was followed by a segment of ileal and sigmoid resection with Jejune-colic anastomosis, which resulted in short bowel syndrome. Resection in the small bowel leads to the worsening of macronutrient limitations, according to the section, largely through changes in size and/or movement of bowel loops. Besides, the ileosigmoid resection can also lead to the loss of the ileocecal valve and the ileal brake, which is the rapid passage of the small intestine [9]. Intestinal adaptation has everything to do with the anatomy that is left after the resection. The presence of an intact ileum and colon is the key. At the same time, the proper function of the ileocecal valve is the second most essential factor that prevents reflux of the colonic content and limits the transit time there. In addition, the colon functions in water absorption, and the process of `carbohydrate salvage` allows the breakdown and absorption of short-chain fatty acids, which improves caloric absorption [10]. The patient possessed both a functional colon and a complete ileocecal valve, most probably leading to successful intestinal adaptation. 

The value of total parenteral nutrition (TPN) in these cases in terms of saving lives and positively influencing physiological functions is not questioned. However, the treatment is high-level, both in terms of the patient's finances and health [11]. Besides the well-defined clinical feature (it should be noted that the mechanism of catheter-related complications in mucous development is not exactly known or described), it can also have both economic and social effects (family expenses, travel limitations). Each case should be individually checked to reach a conclusion on the risk-benefit ratio before a large-volume intestinal resection is implemented [11]. 

## Conclusions

Ileosigmoid knotting (ISK) is a rare but very serious case that requires quick recognition and intervention so that bowel ischemia and gangrene do not happen. The early diagnosis that is made possible by imaging methodologies like contrast-enhanced computed tomography (CECT) and timely actions are indispensable for good outcomes. In cases of confirmed abdominal sepsis, a contrast CT scan might not always be feasible or safe due to the patient's condition or potential risks, so clinicians should depend on clinical evaluation and physical abdominal signs to assess and manage the situation, particularly when ischemic bowel is suspected. The management of the disease requires high fluid resuscitation, the use of an antibiotic for the specific type of anaerobic bacteria present, and surgery as and when needed. For patients with short bowel syndrome, parenteral nutrition and intestinal transplantation are key treatment options, especially for those who qualify and can access these therapies. The choice between treatments should be based on factors such as the patient's response to parenteral nutrition, any complications arising from it, and their overall health condition. Even though short bowel syndrome usually happens after a bowel resection and comes with its own challenges, many patients do well with proper nutritional support and techniques that help the remaining intestine adapt and function better.

## References

[1] Machado NO (2009). Ileosigmoid knot: a case report and literature review of 280 cases. Ann Saudi Med.

[2] Mandal A, Chandel V, Baig S (2012). Ileosigmoid knot. Indian J Surg.

[3] Molla YD, Mequanint MB, Bisrat SH, Workneh GA, Alemu HT (2024). Ileo-ileal knot causing acute gangrenous small bowel obstruction: a case report. J Med Case Rep.

[4] Hirano Y, Hara T, Horichi Y (2005). Ileosigmoid knot: case report and CT findings. Abdom Imaging.

[5] Pattanaik SK, Pattanaik P, Nanda BK (2024). Compound volvulus: ileosigmoid knot. BMJ Case Rep.

[6] Puthu D, Rajan N, Shenoy GM, Pai SU (1991). The ileosigmoid knot. Dis Colon Rectum.

[7] Shepherd JJ (1967). Ninety-two cases of ileosigmoid knotting in Uganda. Br J Surg.

[8] Alsuhaimi MA, Aljaysh MA, Alghamdi HS, Alotaibi MS, Alghamdi AA (2022). A rare cause of acute intestinal obstruction: Ileo-sigmoid knotting type IIA. Ann Med Surg (Lond).

[9] Cohran VC, Prozialeck JD, Cole CR (2017). Redefining short bowel syndrome in the 21st century. Pediatr Res.

[10] Hasosah M, Lemberg DA, Skarsgard E, Schreiber R (2008). Congenital short bowel syndrome: a case report and review of the literature. Can J Gastroenterol.

[11] Morris JA, Selivanov V, Sheldon GF (1983). Nutritional management of patients with malabsorption syndrome. Clin Gastroenterol.

